# Rapid Identification of *Escherichia coli* Colistin-Resistant Strains by MALDI-TOF Mass Spectrometry

**DOI:** 10.3390/microorganisms9112210

**Published:** 2021-10-24

**Authors:** Adriana Calderaro, Mirko Buttrini, Benedetta Farina, Sara Montecchini, Monica Martinelli, Federica Crocamo, Maria Cristina Arcangeletti, Carlo Chezzi, Flora De Conto

**Affiliations:** 1 Department of Medicine and Surgery, University of Parma, Viale A. Gramsci 14, 43126 Parma, Italy; mirko.buttrini@unipr.it (M.B.); benedetta.farina@studenti.unipr.it (B.F.); sara.montecchini@unipr.it (S.M.); federica.crocamo@unipr.it (F.C.); mariacristina.arcangeletti@unipr.it (M.C.A.); carlo.chezzi@unipr.it (C.C.); flora.deconto@unipr.it (F.D.C.); 2 Unit of Clinical Microbiology, University Hospital of Parma, Viale A. Gramsci 14, 43126 Parma, Italy; mmartinelli@ao.pr.it

**Keywords:** colistin resistance, MALDI-TOF MS, Gram-negative bacteria

## Abstract

Colistin resistance is one of the major threats for global public health, requiring reliable and rapid susceptibility testing methods. The aim of this study was the evaluation of a MALDI-TOF mass spectrometry (MS) peak-based assay to distinguish colistin resistant (colR) from susceptible (colS) *Escherichia coli* strains. To this end, a classifying algorithm model (CAM) was developed, testing three different algorithms: Genetic Algorithm (GA), Supervised Neural Network (SNN) and Quick Classifier (QC). Among them, the SNN- and GA-based CAMs showed the best performances: recognition capability (RC) of 100% each one, and cross validation (CV) of 97.62% and 100%, respectively. Even if both algorithms shared similar RC and CV values, the SNN-based CAM was the best performing one, correctly identifying 67/71 (94.4%) of the *E. coli* strains collected: in point of fact, it correctly identified the greatest number of colS strains (42/43; 97.7%), despite its lower ability in identifying the colR strains (15/18; 83.3%). In conclusion, although broth microdilution remains the gold standard method for testing colistin susceptibility, the CAM represents a useful tool to rapidly screen colR and colS strains in clinical practice.

## 1. Introduction

Antimicrobial resistance is one of the major threats for global public health, since many pathogens are developing resistance mechanisms to almost all currently available antimicrobial drugs [1,2,3]. This phenomenon is mostly related to the misuse and overuse of antimicrobials, which led to the emergence of multidrug-resistant (MDR), extensively-drug-resistant (XDR) and pan-drug-resistant bacteria [4,5,6,7]. In particular, infections caused by resistant Gram-negative bacteria, such as *Enterobacteriaceae*, *Pseudomonas aeruginosa* and *Acinetobacter baumannii,* are a broad matter of concern, because of the ineffectiveness of conventional treatments and the lack of new antimicrobial agents against them [1,3]. Therefore, the occurrence and spread of resistant bacterial strains prompted the re-evaluation of polymixins (colistin and polymixin B), an old class of cationic, cyclic-polypeptide antibiotics [6,8], whose clinical use was previously limited for their reported nephrotoxicity and neurotoxicity [9,10,11]. To date, colistin is considered a “last resort” antibiotic, namely a valid alternative to the classic antimicrobial agents ineffective against MDR Gram-negative pathogens [1,3,6,8]. Given its saving role against the life-threatening MDR and XDR bacterial infections, colistin was largely and recklessly employed both in human and veterinary medicine, resulting in the emergence of colistin-resistant pathogens, mainly Gram-negative bacteria [1,3,6,12]. Commonly, colistin-superbugs are the phenotypic expression of regulatory or mutational events in chromosomal genes [13,14,15]. However, resistance to this antibiotic can also be acquired by a plasmid-mediated strategy involving the mobile colistin resistance (*mcr*) genes (*mcr1-mcr10*) [3,16,17,18,19]. The discovery of the *mcr* genes on mobile genetic elements raised alarm given the possibility of their rapid dissemination by horizontal transfer [19,20]; therefore, reliable methods to detect colistin resistance are urgently needed [21,22,23]. Among the different laboratory techniques for testing the colistin susceptibility, the phenotypic ones, such as the disc diffusion and the gradient tests, are not very adequate because of the long turnaround time required, the low *mcr* sensitivity and specificity and the poor diffusion in agar of this drug [24,25]. Thus, the European Committee on Antimicrobial Susceptibility Testing (EUCAST) and the Clinical and Laboratory Standards Institute (CLSI) recommend the use of the broth microdilution (BMD), as the reference method to test colistin susceptibility among Gram-negative bacteria [25,26]. Even if BMD is considered the gold standard technique in determining the minimal inhibitory concentration (MIC) values of colistin in clinical microbiology laboratories, it is laborious and time consuming [24,25]. On the other hand, faster tools than BMD such as the automated MIC-determining systems (i.e., MicroScan, Sensititre, MICRONAUT-S, BD Phoenix, Vitek 2) do not meet the recommendations of EUCAST and CLSI, since they show low agreement with the reference test and, additionally, high rates of false susceptibility results [22,24,27]. Nevertheless, reliability and rapidity in detection of colistin resistance are crucial for antimicrobial stewardship and could be achieved by fast and reliable methods, such as MALDI-TOF MS [28]. This technique is already employed in clinical microbiology laboratories for the phenotypic identification of bacterial and fungal strains and its potentialities in predicting the antimicrobial resistance are being studied [26,29,30]. In particular, the MALDI-TOF MS approach for testing polymixins-resistance is based on the detection of biomarkers associated with the modified lipid A, which is the phenotypic result of both chromosomal and plasmid encoded resistance to colistin in Gram-negative bacteria. Therefore, given the inherent negative charge of the lipid A, several studies aimed to create MALDI-TOF MS tests to screen colistin resistance in Gram-negative bacteria by operating in a negative ion mode of the mass spectrometer [28,31,32,33]. However, to date, the negative ion mode is not currently and widely available on diagnostic routine mass spectrometers, since it works in a molecular mass range different from that used for the bacterial and fungal identification [32]. In this study, we describe an alternative approach for the identification of colistin resistance in Gram-negative bacteria by proposing a MALDI-TOF MS protein peak-based assay developed on the basis of spectra acquired in a positive linear mode embedded in the most widely used MALDI-TOF MS instrument available in clinical microbiology laboratories. The main aim of the study was to create a classifying algorithm model (CAM) able to rapidly detect and identify the colistin-resistant strains in clinical practice, in order to shorten the turnaround time by a simple and inexpensive tool.

## 2. Materials and Methods

### 2.1. Bacterial Strains

A total of 104 Gram-negative bacteria were included in this study: 71 *Escherichia coli* (Ec) strains (53 and 18 of human and veterinary origin, respectively) and 33 control other than *E. coli* strains, 15 *Klebsiella pneumoniae* (1 of animal origin), 9 *Pseudomonas aeruginosa*, 4 *Acinetobacter baumanii*, 5 *Achromobacter xylosoxidans*. The human strains were collected at the Unit of Clinical Microbiology of the University Hospital of Parma (81 strains) and at the Unit of Microbiology and Virology of the Hospital of Piacenza (4 strains) and the animal strains at the Department of Veterinary Science of Parma (Italy).

### 2.2. Colistin Susceptibility Testing

Colistin minimal inhibitory concentration (MIC) was determined by BMD (Liofilchem, Roseto degli Abruzzi, Teramo, Italy), following the manufacturer’s instructions. Results were interpreted using Clinical and Laboratory Standards Institute (CLSI) breakpoints.

### 2.3. MALDI-TOF MS Protein Peak-Based Assay

For testing colistin resistance by MALDI-TOF MS, a protein extraction protocol was performed. All the bacterial strains were cultured on horse blood agar (Kima, Italy) plates and incubated at 37 °C for 24 h; then, the isolated colonies were used to obtain a 3 McFarland bacterial suspension in sterile double-distilled water. An aliquot of 300 µL of the bacterial suspension was added to 900 µL of absolute ethanol, homogenized by vortex for 20 s and then centrifuged at 14.000× *g* for 2 min. The supernatant was discharged, and the pellet was dried for 5 min under a laminar flow cabinet at room temperature, then suspended in 15 µL of 70% formic acid and 15 µL of acetonitrile, and finally vortexed (20 s) and centrifuged (14.000× *g* for 2 min). One µL of this supernatant obtained by protein extraction protocol was transferred on a MALDI-TOF target plate (10 replicates for each strain), dried under a stem of air and then overlaid with 1 µL of α-Cyano-4-hydroxycinnamic acid Matrix (HCCA-Bruker Daltonics, Bremen, Germany), solubilized in 30:70 (*v*:*v*) acetonitrile/trifluoroacetic acid 0.01% (TA30 Organic Solvent). The dried spots were analysed by the Autoflex Speed mass spectrometer (Bruker Daltonics, Germany), previously calibrated with “Bruker Bacterial Test Standard” according to manufacturer’s instructions and set in MBT_ Standard method (positive linear mode, with 60 Hz laser frequency, ion source voltage 20 kV and mass molecular range 2–20 kDa). The spectra acquisition was performed in different independent experiments, by different operators in different days, in manual mode, in different points of the well with a laser intensity ranging from 30 to 40%, and an overall 1400 laser-shot, by 200 shot steps. The acquired spectra were baselined and smoothed by FlexAnalysis software (version 3.1, Bruker Daltonics), and only those with >104 intensity arbitrary units were used for further analysis. The normalized spectra were then loaded into the MALDI Biotyper software (version 3.1.66, Bruker Daltonics) to verify their validity, and to identify the bacterial species; the replicates identified with a <2-score value were discarded. 

### 2.4. Classifying Algorithm Model

Based on BMD susceptibility results, the 71 *E. coli* strains were divided in two groups: “training set” and “test set”. The “training set” included 10 *E. coli* strains, 5 colistin-resistant (colR-Ec) and 5 colistin-susceptible (colS-Ec), randomly selected; the “test set” included the remaining 61 *E. coli* strains.

To rapidly and correctly identify both the colistin-resistant (colR) strains and the susceptible (colS) ones, a classifying algorithm model (CAM) was developed by using the “training set”. A total of 80 “training set” spectra (8 replicates/strain) were imported in ClinProTools software (version 3.0, Bruker Daltonics), in order to develop a CAM able to discriminate between the colR-Ec and the colS-Ec on the basis of their different protein profiles. The analysis was performed focusing on the molecular mass range 2–20 kDa with a 4 signal-to-noise threshold, an 0.08 relative threshold base peak, and the “Shift Maximum Peak” parameter set up at 1000 parts per million (ppm). The software calculated an average spectrum of each isolate (single average spectrum), an average spectrum of both the classes considered (colR-Ec average spectrum and colS-Ec average spectrum) and an average spectrum based on all replicates of all analyzed isolates (cumulative average spectrum), and then provided a list of peaks potentially differentiating the colR-Ec strains and the colS-Ec ones. In addition, this spectra dataset (“training set”) was analysed by Principal Component Analysis (PCA), an unsupervised hierarchical type of clustering, in order to visualize the homogeneity and heterogeneity of the protein spectra. The PCA results are called scores and are derived and displayed in various plots. The score output represents the original data mapped into the new coordinate system, which is defined by the Principal Components (PCs). Within the score plot, outlier spectra from a group or from several groups can be discovered and visualized. The outliers are spectra that are extreme or do not fit the PCA model. Independently from the PC coordinates, the score plots contain the same spectra number as the original data set. Moreover, the percentage of the “explained variance” of the single given PC was also reported.

For the development of the CAM, the Genetic Algorithm (GA), the Quick Classifier (QC), and the Supervised Neural Network (SNN) algorithm-based models were applied to the preliminary pattern of protein peaks found by the software. Each algorithm performed a selection of different discriminating peaks in order to improve its classification performance. Each CAM was characterized by recognition capability (RC) and cross-validation (CV) values, parameters of the accuracy of the model. For the development of each algorithm, different combinations of parameters were evaluated and, for each one, the most performing in terms of RC and CV was reported. The reliability and the accuracy of each algorithm-based model were verified by performing an internal and an external validation. In particular, the “training set” were used for the internal validation, while the external validation was achieved with the “test set”.

#### 2.4.1. Genetic Algorithm

The Genetic Algorithm (GA) works on a population of peaks and selects the fittest peaks combinations that are the most relevant for the classification. At each step, the GA randomly selects peaks from the preliminary pattern of protein peaks found by the Software and uses them as “parents” to produce the “children” for the next generation. In this study, the parameters of this algorithm were set as follows. A maximum number of 15 peaks was chosen to be included in the model, with an automatic detection mode to determine the best number of peak combinations to integrate (npc) by applying the heuristic formula NPC = 100 + (Number of picked peaks × 20)/(Maximal number of peaks in model + 1). Within the GA algorithm, the k-nearest neighbor (k-NN) classifier algorithm allowed us to obtain the final classification. The k-NN algorithm calculated the distances between the points in the n-dimensional space; each point corresponded to a spectrum whose area defined its coordinates. The number of neighbors, the mutation rate and the crossover rate were set at 7 (values among 3, 5, and 7), 0.2 (range values 0.0–1.0) and 0.5, respectively. In ClinProTools, both the mutation rate and the crossover rate are advanced parameters. In particular, the mutation rate is the probability of a mutation, namely the random exchange of a peak within peak combinations by a randomly selected new one; on the other hand, the crossover rate is the likelihood of a crossover between peaks combinations.

#### 2.4.2. Quick Classifier

The QuickClassifier (QC) is a univariate sorting algorithm. The class averages of the peak areas are stored in the model, together with statistical data such as the *p*-values at certain peak positions. For classification, the peak areas/intensities are sorted per peak and a weighted average over all peaks is calculated.

The QC automatically uses automatic peak detection to determine the best number of peaks to be integrated in the model (maximum is 25 peaks). The sort mode used for peak ranking and as weight was *p*-value obtained by Student’s *t*-test/ANOVA.

#### 2.4.3. Supervised Neural Network

The Supervised Neural Network (SNN) is a prototype-based classification algorithm that identifies some characteristic spectra for each class, which could be consider as prototypes of that class. In an initial step, a predefined number of prototypes are spread over the data space by using the Batch-Neural-Gas algorithm, which gives an optimal distribution of the prototypes over the data space in accordance with the data density properties. In a second step, the SNN optimizes the positions of the prototypes with respect to the class information (supervised) minimizing the empirical risk. Thereby, the used metric of the data space is adapted such that dimensions, which are relevant for the class separation, are higher weighted than dimensions that do not contribute to class separation. This procedure of optimizing prototype positions with a combined feature selection is applied iteratively for a predefined upper limit of steps. 

The SNN uses an automatic peak detection mode to determine the best number of peaks (maximum 25 peaks) to be integrated in the model and the automatic detection of prototype number was applied. The upper limit of cycles to run for optimizing the prototype positions was set at 2000.

### 2.5. Statistical Analysis 

Statistical Analysis was automatically performed by ClinProTools software. In particular, the *p*-value is obtained by both parametric and non-parametric statistical tests. With regards to parametric tests, the software automatically selected between Student’s *t*-test (two classes involved) or ANOVA (more than two classes involved). Similarly, for non-parametric tests, the software automatically selected between Wilcoxon test (2 classes involved) or Kruskal–Wallis test (more than two classes involved).

The *p*-value was calculated by comparing each single average spectrum with the cumulative average spectrum, based on both parametric (Student’s *t*-test) and non-parametric (Wilcoxon—W) statistical tests. The software considered the peaks associated to a *p*-value ≤ 0.05 as potentially able to discriminate between the considered classes. However, since the best discriminating peaks are associated to low *p*-values, the significance threshold was set to 0.01, in order to improve the performance.

## 3. Results

**Figure 1:**
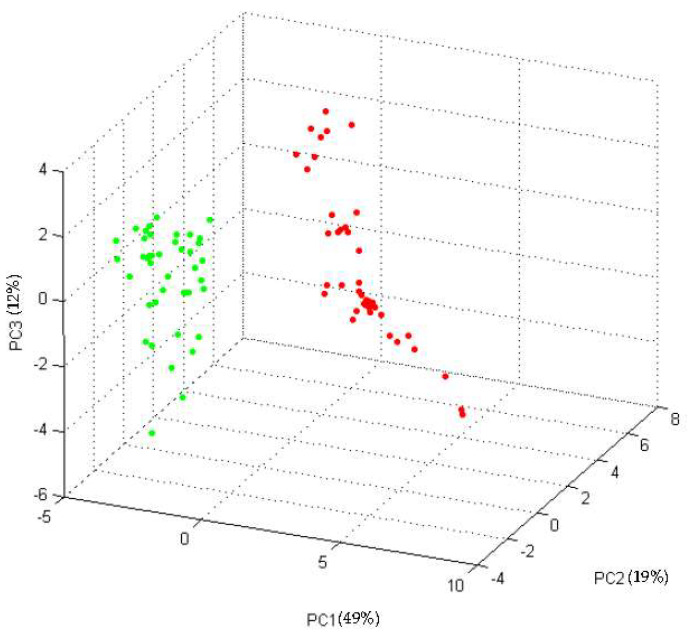
Three-dimensional plot of the spectra of the “training set” strains obtained by Principal Component Analysis (PCA) (dots of the same colour represent replicates of strains of the same class: colR-Ec strains in red and colS-Ec strains in green). For each principal component (PC), the percentage of the “explained variance” is reported in round bracket.

According to the colistin susceptibility testing results, among all *E. coli* isolates, 48 (33 human and 15 animal strains) were colistin-susceptible and 23 (20 human and three animal strains) were colistin-resistant (MIC ≥ 4 mg/L). Both the colR-Ec strains and the colS-Ec strains were correctly identified at the species level by MALDI-TOF MS, with score values above 2.0. When the “training set” was used to develop the CAM, a list of 47 potential discriminating peaks (Supplementary Table S1) was obtained by the ClinProTools software. The additional PCA analysis of the spectra showed two different clusters referred to colR-Ec and colS-Ec strains (Figure 1).

**Figure 2:**
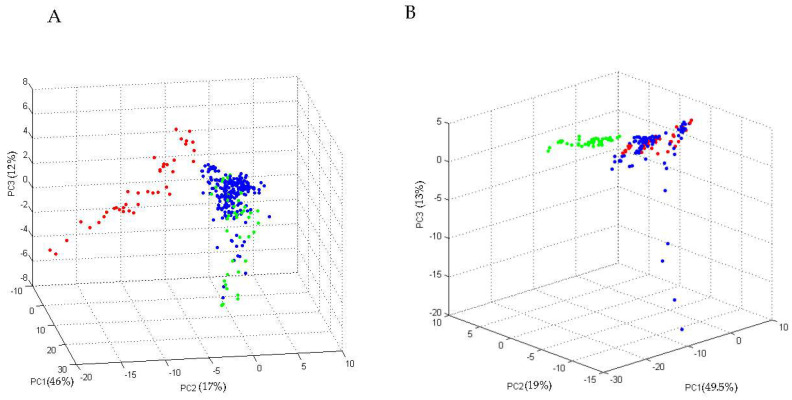
Three-dimensional plot of the spectra of the “training set” strains obtained by Principal Component Analysis (PCA) (colR-Ec strains in red and colS-Ec strains in green) in comparison to: (**A**) colS-Ec “test set” strains (blue) and (**B**) colR-Ec “test set” strains (blue) (dots of the same colour represent replicates of strains of the same class). For each principal component (PC), the percentage of the “explained variance” is reported in round bracket.

In order to verify the reliability of these discriminating peaks, 61 different strains (“test set”), 43 colS-Ec and 18 colR-Ec were loaded into ClinProTools software and the PCA was created (Figure 2A,B, respectively).

The “PCA-3D plot” obtained for colR-Ec showed that these strains clustered close to colR-Ec strains used as “training set” and were totally separated from colS-Ec strains. Similarly, the PCA obtained for colS-Ec strains showed that these strains clustered close to colS-Ec strains used as “training set” and were totally separated from colR-Ec strains.

The three algorithms applied to the pattern of 47 peaks showed different performances in terms of RC, CV and list of peaks involved, as reported in Table 1.

The GA-based model automatically selected seven peaks with a *p*-value < 0.008 for both statistical tests. Both the RC and CV values were 100%. As concern the internal and the external validations, this CAM correctly classified 100% and 82% (50/61) of the *E. coli* strains included in the “training set” and in the “test set”, respectively. In particular, 88.9% (16/18) of colR-Ec and 79% (34/43) of the colS-Ec strains of the “test set” were correctly classified (Table 2).

The SNN-based model automatically selected seven peaks associated to a *p*-value < 0.000001 for both statistical tests, with 100% and 97.62% of RC and CV, respectively. Concerning the internal validation, 100% of the colR-Ec and colS-Ec “training set” strains were correctly classified (Table 2); conversely, this CAM correctly classified 93.4% of the strains included in the “test set” (57/61): 83.3% (15/18) and 97.7% (42/43) of the colR-Ec and colS-Ec external control strains, respectively, were correctly classified (Table 2).

Finally, the QC-based model automatically selected three peaks with a *p*-value < 0.000001 for both statistical tests, classifying strains with overall RC (98.81%) and CV (94.21%) values lower than the other two algorithm-based models tested. The QC-based CAM correctly classified 100% of the internal control strains and 73.8% of the strains included in the “test set” (45/61): 94.4% (17/18) of the colR-Ec and 65.1% (28/43) of the colS-Ec test strains, respectively, were correctly classified (Table 2). 

With regard to the absolute number of errors, GA-based CAM failed to classify 11 strains out of the 61 included in the “test set”, while SNN-based CAM failed to classify four strains, and QC-based CAM 16.

Among the 18 colR-Ec strains, one human strain was wrongly classified as colS-Ec by each CAM tested; similarly, among the 43 colS-Ec strains, one animal strain was wrongly classified as colR-Ec by the three CAMs.

**Figure 3:**
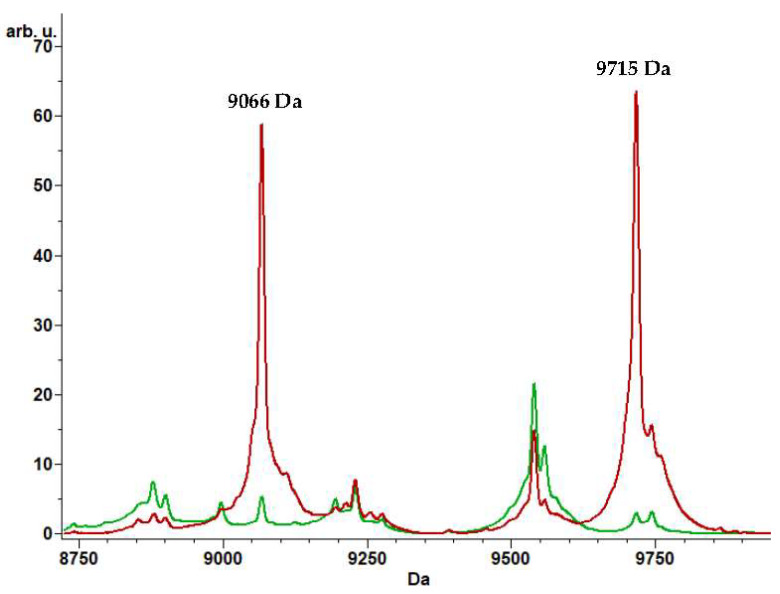
Peaks used for the classification by the SNN-based CAM (9066 Da and 9715 Da) not involved by the GA and QC algorithms (colR-Ec “training set” Average spectrum in red and colS-Ec “training set” Average spectrum in green). arb. u. arbitrary units; Da dalton.

The pattern of peaks used for the classification by the SNN-based CAM included two peaks (9066 Da and 9715 Da) (Figure 3), not included in the GA and QC algorithms’ list of peaks.

The molecular weights of these two peaks were found to correspond to those of a hypothetical protein related to the IncK2 carrying mcr-1 gene plasmid (Accession number ASO65104.1; 86 amino acids; molecular weight 9090 Da) and of a transcriptional regulator linked to a Plasmid-mediated mcr-1 (pICBEC7Pmcr) in a carbapenem-susceptible *E. coli* strain (Accession number OKO56538.1; 85 amino acids; molecular weight 9700 Da), respectively.

These three different CAMs patterns of peaks were evaluated also with colR and colS strains (16 and 17 strains, respectively) other than *E. coli* (Table 3). GA-based CAMs correctly classified 45.5% (15/33) of strains: 50% (8/16) of colR and 41.2% (7/17) of colS strains. SNN-based CAM correctly classified 48.5% (16/33) of strains: 50% (8/16) of colR and 47% (8/17) of colS strains. QC-based CAM classified all strains as colR.

## 4. Discussion

In the last decade, colistin has been proposed as a last-line antibiotic for the treatment of MDR Gram-negative infections.

However, despite its high capacity in bacterial killing, colistin resistance phenomenon is emerging as a result of mutations in the efflux pump operon, and in genes encoding lipid A. These resistance mechanisms to colistin have gained global attention and pose a new threat to public health [26,33].

Furthermore, as widely reported by EUCAST and CLSI, the methods most frequently used in clinical microbiology laboratories for performing antimicrobial susceptibility testing (automated systems, E-test) are not reliable for detecting colistin resistance, concluding that BMD represents the reference method for colistin susceptibility testing.

More rapid, and cost-effective clinical microbiology technologies, such as MALDI-TOF MS, are currently being evaluated in order to overcome the use of BMD method and to obtain a reliable detection of colistin-resistant strains, by directly assessing the biochemical cause of resistance, the modification of lipid A [31]. However, the MALDI-TOF MS method for colistin resistance detection is usually performed in a linear negative-ion mode [31], not embedded in all instruments used in diagnostic laboratories, in a mass range < 2 kDa. This study aimed to detect colistin resistance in *E. coli* strains by MALDI-TOF MS in the linear positive-ion mode, within the mass range 2-–20 kDa commonly used for the routine identification of bacteria and fungi [34,35,36,37]. Firstly, we created a CAM to automatically distinguish *E. coli* strains as colR or colS using 10 *E. coli* strains (5 colR and 5 colS), arbitrarily selected. The multivariate unsupervised statistical analysis, PCA, applied to this data set, showed the spectra replicates of the two different analyzed classes (colistin-resistant and colistin-susceptible) placed in two well separated clusters, suggesting the possible differentiation of these two classes based on the presence of specific peaks in the mass range 2–20 kDa. In particular, a pattern of 47 potential discriminating peaks was evaluated using three different algorithms-based CAMs (GA, SNN and QC). The three CAMs identified different patterns of discriminating peaks, which, however, showed similar RC (100%, 100% and 98.81%, respectively), and CV (100%, 97.62% and 94.21%, respectively) values. In addition, in all three cases a correct identification of the 100% of the internal control strains was obtained. Despite that GA-based CAM showed the highest values of RC and CV, it demonstrated a moderate ability to detect colS-Ec strains (34/43; 79%). On the contrary, the external validation of the QC-based CAM was not satisfactory, since only 73.8% (45/61) of the strains was correctly identified, and in particular only 65.1% (28/43) of colS-Ec strains.

Among the three CAMs tested, even if it showed the lowest ability in identifying colR-Ec strains (15/18; 83.3%), the SNN-based model correctly identified the greatest number of colS-Ec strains (42/43; 97.7%). The only colS-Ec strain wrongly classified by this CAM was also mis-identified by the other two.

However, if considering the absolute number of errors, GA-based CAM failed to classify 11 strains of the validation set, while SNN-based CAM failed to identify only four strains. The correct identification of the majority of the colS-Ec strains by SNN-based CAM could be due to the inclusion in the classification peak list of two peaks (9066 Da and 9715 Da) having a molecular weight similar to the two proteins related to a plasmid, which carries mcr-1 gene found in *E. coli*. The same reason could explain the failure of the same CAM in the classification of three colR strains, which may show a colistin resistance mechanism different from mcr-1.

Each CAM showed a limited capability in classifying Gram-negative colR and colS strains other than *E. coli*, likely due to the diversity between their species-specific protein profiles and those of *E. coli*, on which the development of the three CAMs was based. Therefore, a classification model is able to perform an intraspecific discrimination between colR and colS strains, which are probably classified on the basis of species-specific colistin resistance mechanisms, whose molecular effectors could have a molecular mass within the 2–20 kDa range. Although this approach is not based on the traditional colistin resistance biomarkers (i.e., modifications in lipid A, involving phosphoethanolamine), the development of a species-specific CAM allows a rapid screening of colR and colS strains on the basis of species-related discriminating peaks, and without resorting to the more time-consuming BMD method. As a matter of fact, although the conventional antibiotic susceptibility tests, such as BMD, allow the phenotypic characterization of resistant strains, they suffer from a higher time-to-result because of the long time required for the bacterial growth and the phenotypic expression of the resistance mechanism, consequently preventing a prompt and appropriate treatment.

## 5. Conclusions

In conclusion, the present study describes an alternative, rapid, simple to perform, inexpensive and reliable MALDI-TOF MS application for the identification of colR *E. coli* strains in routine diagnostic. Although no one of the CAM created in this study is 100% reliable for both the categories tested, this approach could represent an alert, at the same time as the bacterial identification, for the presence of colR strains, which, as suggested by CLSI and EUCAST, should be investigated using the gold standard method (BMD assay) for MIC determination or molecular approach to determine genetic resistance mechanisms.

## Figures and Tables

**Table 1:** Performances and list of peaks of the different algorithms tested. GA Genetic Algorithm; SNN Supervised Neural Network; QC Quick Classifier; RC Recognition Capability; CV Cross Validation; Da Dalton; + peak involved in classifying algorithm model (CAM).

Algorithms Model	RC (%)	CV (%)	Peaks Used for Classification (Da)											
			4177	4365	4440	4449	4498	5612	6257	6283	8330	8878	9066	9715
GA	100	100	+	+	+		+		+		+	+		
SNN	100	97.62	+		+			+	+	+			+	+
QC	98.81	94.21				+				+		+		

**Table 2:** Classification of the internal and external validation *E. coli* strains by the 3 different classifying algorithm models (CAMs). BMD Broth microdilution; GA Genetic Algorithm; SNN Supervised Neural Network; QC Quick Classifier; colR colistin-resistant; colS colistin-susceptible.

Set of Strains	BMD	No.	CAM Classification								
			GA			SNN			QC		
			colR	colS	Correctly Classified (%)	colR	colS	Correctly Classified (%)	colR	colS	Correctly Classified (%)
Internal validation	colR	5	5		5/5 (100)	5		5/5 (100)	5		5/5 (100)
	colS	5		5	5/5 (100)		5	5/5 (100)		5	5/5 (100)
		10			10/10 (100)			10/10 (100)			10/10 (100)
External validation	colR	18	16	2	16/18 (88.9)	15	3	15/18 (83.3)	17	1	17/18 (94.4)
	colS	43	9	34	34/43 (79)	1	42	42/43 (97.7)	15	28	28/43 (65.1)
		61			50/61 (82)			57/61 (93.4)			45/61 (73.8)
Overall		71			60/71 (84.5)			67/71 (94.4)			55/71 (77.5)

**Table 3:** Classification of strains other than *E. coli* by the 3 different classify algorithm models (CAMs). BMD Broth microdilution; GA Genetic Algorithm; SNN Supervised Neural Network; QC Quick Classifier; colR colistin-resistant; colS colistin-susceptible.

Strains	BMD	No.	CAM Classification								
			GA			SNN			QC		
			colR	colS	Correctly Classified (%)	colR	colS	Correctly Classified (%)	colR	colS	Correctly Classified (%)
*K. pneumoniae*	colR	7	3	4	3/7 (42.9)		7	0/7 (0)	7		7/7 (100)
*P. aeruginosa*	colR	2	2		2/2 (100)	2		2/2 (100)	2		2/2 (100)
*A. baumannii*	colR	2	2		2/2 (100)	2		2/2 (100)	2		2/2 (100)
*A. xylosoxydans*	colR	5	1	4	1/5 (20)	4	1	4/5 (80)	5		5/5 (100)
		16			8/16 (50)			8/16 (50)			16/16 (100)
*K. pneumoniae*	colS	8	1	7	7/8 (87.5)		8	8/8 (100)	8		0/8 (0)
*P. aeruginosa*	colS	7	7		0/7 (0)	7		0/7 (0)	7		0/7 (0)
*A. baumannii*	colS	2	2		0/2 (0)	2		0/2(0)	2		0/2 (0)
		17			7/17 (41.2)			8/17 (47)			0/17 (0)
Overall		33			15/33 (45.5)			16/33 (48.5)			16/33 (48.5)

## Data Availability

The data presented in this study are available in the manuscript and in the supplementary materials (Supplementary Table S1).

## Supplementary Materials

The following is available online at https://www.mdpi.com/article/10.3390/microorganisms9112210/s1 Table S1: list of potential discriminating peaks.
