# Supplementary material for: Rapid Identification of Escherichia coli Colistin-Resistant Strains by MALDI-TOF Mass Spectrometry
Source: Microorganisms. 2021 Oct 24;9(11):2210. doi: 10.3390/microorganisms9112210 (PMC8623207; doi:10.3390/microorganisms9112210)
Supplement: Supplementary file 1 [file microorganisms-09-02210-s001.zip › microorganisms-1405968-supplementary.pdf]

Supplementary Table S1: list of potential discriminating peaks. *t*-test: *p*-value obtained by Student's *t*-test; W-test: *p*- value obtained by Wilcoxon test; Ave: the peak area/intensity average value; colR-Ec: colistin-resistant *E. coli* strains; colS-Ec: colistin-susceptible *E. coli* strains; SD: Standard Deviation.

Mass	*t*-test	W-test	Ave colR-Ec ± SD	Ave colS-Ec ± SD
2181	< 0.000001	< 0.000001	2.72 ± 1.3	5.91 ± 3.24
2690	< 0.000001	0.00000211	7.18 ± 4.04	15.26 ± 8.45
3128	< 0.000001	< 0.000001	4.3 ± 2.07	10.08 ± 5.15
3158	< 0.000001	< 0.000001	4.2 ± 2.15	9.52 ± 5.16
3638	< 0.000001	< 0.000001	8.71 ± 3.2	14.46 ± 4.22
3936	< 0.000001	< 0.000001	2.2 ± 1.05	4.28 ± 1.4
4177	< 0.000001	< 0.000001	8.98 ± 3.56	3.91 ± 1.69
4365	< 0.000001	< 0.000001	9.54 ± 5.78	26.55 ± 9.74
4383	< 0.000001	< 0.000001	1.93 ± 1.09	4.49 ± 1.36
4440	< 0.000001	< 0.000001	4.4 ± 1.48	9.73 ± 2.19
4449	< 0.000001	< 0.000001	4.01 ± 1.33	8.27 ± 1.66
4498	< 0.000001	< 0.000001	3.86 ± 0.59	6.56 ± 2.42
4534	< 0.000001	< 0.000001	28.53 ± 18.44	3.22 ± 1.81
4597	0.00000326	0.00000141	4.02 ± 1.06	5.57 ± 1.62
4615	0.00038	0.00234	9.76 ± 3.41	7.45 ± 1.66
4858	< 0.000001	< 0.000001	46.79 ± 29.29	3.4 ± 2.62
5071	< 0.000001	< 0.000001	5.98 ± 1.38	8.38 ± 2.08
5098	0.000773	0.00314	11.54 ± 7.74	17.79 ± 8.14
5152	< 0.000001	< 0.000001	5.63 ± 2.61	10.68 ± 3.7
5238	< 0.000001	< 0.000001	7.12 ± 4.52	1.31 ± 0.46
5329	0.000489	0.000863	5.95 ± 1.3	5 ± 0.97
5348	< 0.000001	< 0.000001	5.01 ± 1.45	8.23 ± 1.12
5383	< 0.000001	< 0.000001	20.72 ± 11.75	44.64 ± 14.5
5462	0.00000313	0.00000368	4.21 ± 2.06	2.26 ± 1.17
5612	< 0.000001	< 0.000001	6.98 ± 2.27	3.76 ± 1.3
5754	< 0.000001	0.00253	8.24 ± 7.16	1.49 ± 0.48
6257	< 0.000001	< 0.000001	10.58 ± 6.73	29.57 ± 8.35
6283	< 0.000001	< 0.000001	2.88 ± 1.65	7.34 ± 1.47
6318	< 0.000001	< 0.000001	7.54 ± 4.64	19.84 ± 5.87
6388	< 0.000001	< 0.000001	3.04 ± 1.38	6.94 ± 1.79
6413	< 0.000001	< 0.000001	3.39 ± 1.21	9.95 ± 3.69
6487	< 0.000001	< 0.000001	2.1 ± 0.98	4.46 ± 1.16
7276	< 0.000001	< 0.000001	13.04 ± 6.52	31.98 ± 7.24
7301	< 0.000001	< 0.000001	2.5 ± 0.94	5.85 ± 2.11
7335	< 0.000001	< 0.000001	2 ± 0.73	9.46 ± 6.72
7872	< 0.000001	< 0.000001	2.5 ± 1.24	4.96 ± 1.06
8330	0.000164	0.00847	7.3 ± 6.12	3.14 ± 0.76
8352	0.00000268	< 0.000001	9.22 ± 5.35	4.42 ± 1.44
8371	0.000284	0.00112	4.84 ± 3.05	7.14 ± 2.22
8856	< 0.000001	< 0.000001	2.43 ± 0.92	4.84 ± 1.31
8878	< 0.000001	< 0.000001	3.28 ± 1.09	7.72 ± 1.57
8899	< 0.000001	< 0.000001	2.65 ± 0.95	5.89 ± 1.63
9066	< 0.000001	< 0.000001	59.25 ± 28.87	5.65 ± 3.81
9538	< 0.000001	< 0.000001	15.22 ± 5.55	21.91 ± 4.14
9715	< 0.000001	< 0.000001	65.24 ± 34.86	3.99 ± 3.1
9740	< 0.000001	< 0.000001	17.39 ± 5.51	3.74 ± 6.12
10302	< 0.000001	< 0.000001	1.89 ± 1.24	4.8 ± 1.29
